# Comparison of magnetic resonance imaging and computed tomography for bone assessment of neurogenic heterotopic ossification of the hip: a preliminary study

**DOI:** 10.1186/s13018-021-02869-6

**Published:** 2021-12-20

**Authors:** Raphaël Amar, Tristan Thiry, Marjorie Salga, Dominique Safa, Annaëlle Chetrit, Laure Gatin, Robert-Yves Carlier

**Affiliations:** 1 Department of Radiology, Assistance Publique - Hôpitaux de Paris (AP-HP), DMU Start Imaging, Raymond Poincaré Teaching Hospital, Garches, France; 2 Department of Physical Medicine and Rehabilitation, Assistance Publique - Hôpitaux de Paris (AP-HP), Raymond Poincaré Teaching Hospital, CIC 1429, Garches, France; 3 UVSQ, INSERM U1179, END-ICAP, University of Paris-Saclay, Montigny-le-Bretonneux, France; 4 Group for Research in Neuro-Orthopedics From Garches (GRENOG), Garches, France; 5 Department of Orthopaedic and Trauma Surgery, Assistance Publique - Hôpitaux de Paris (AP-HP), Raymond Poincaré Teaching Hospital, Garches, France

**Keywords:** Neurogenic heterotopic ossification, Magnetic resonance imaging, Zero Echo Time

## Abstract

**Background:**

Neurogenic heterotopic ossification (NHO) is a frequent complication, often involving the hip. The functional impact may require surgical management and pre-surgical imaging assessment is necessary, usually by computed tomography (CT). We aimed to compare the performances of magnetic resonance imaging (MRI) and CT for bone assessment on pre-surgical imaging of the heterotopic ossifications and their features in NHO of the hip.

**Methods:**

This single-center prospective preliminary study included all patients who underwent surgery for NHO with joint limitation from July 2019 to March 2020. All patients had a CT after biphasic iodinated solution injection and an MRI including T1-weighted, STIR and ZTE sequences. Standardized reports were completed for both exams for each patient, evaluating location, implantation and fragmentation of NHO, relation to the joint capsule and bone mineralization, then were compared.

**Results:**

Seven patients from 32 to 70 years old (mean = 50.2 ± 17.2 years) were evaluated. NHO were bilateral in 2 patients, for a total of nine hips: six right hips and three left hips. Observed concordance rates between MRI and CT were, respectively, 94.4% for location, 100% for circumferential extension, 87.3% for implantation 88.9% for fragmentation, 77.8% for relation to the joint capsule and 66.7% for bone mineralization. It was 100% for femoral neck fracture and osteonecrosis of the femoral head.

**Conclusion:**

This preliminary study suggests that pre-surgical MRI imaging should be considered as effective as CT for bone assessment of NHO and their features.

*Trial registration*: ClinicalTrials.gov, NCT03832556. Registered February 6, 2019, https://clinicaltrials.gov/ct2/show/NCT03832556.

## Background

Neurogenic heterotopic ossification (NHO) is a frequent complication of central nervous system injuries [1] or after total hip arthroplasty [2], often involving the hip.

Surgical management of NHO is complex and requires a team of specialized surgeons [3]. Pre-surgical imaging assessment is necessary to prevent intra-operative and post-surgical complications, such as hemorrhages or fractures, and allows decreasing these risks.

Computed Tomography (CT) is currently the gold-standard pre-surgical imaging assessment, providing high resolution and excellent contrast for vessels thanks to iodinated contrast injection [4]. Heterotopic ossifications cartography and evaluation of their relations to vessels and nerves can be performed [5].

Magnetic resonance imaging (MRI) is a useful exam at the early stage of development of the NHO, evidencing a heterogeneous hyperintensity on short-tau inversion recovery (STIR) images [6] with peripheral contrast enhancement on contrast-enhanced T1-weighted fat-saturated sequence [7], and allowing to eliminate differential diagnoses such as haematoma, infection or, more theoretically because of the clinical context, osseous tumor. However, to our knowledge, its use for pre-surgical imaging assessment has not been evaluated yet. A Zero Echo Time (ZTE) sequence with excellent osseous contrast [8] was developed a few years ago. Its interest has already been proved for evaluation of menisci and articular cartilage [9, 10], but also for imaging of the shoulder [11] and evaluation of cervical neural foraminal stenosis [12]. That is why MRI seems to be able to allow heterotopic ossifications analysis equivalent to a CT scan.

We aimed to compare the performances of MRI and CT to assess heterotopic ossifications locations, their features, and qualitative evaluation of bone mineralization on pre-surgical assessment of NHO of the hip.

## Materials and methods

This single-center prospective preliminary study included all consecutive patients who underwent surgery for symptomatic NHO of the hip in our hospital from July 2019 to March 2020. Institutional review board approval was obtained and all patients gave informed consent to participate.

### Inclusion criteria

All patients over 18 years old followed for NHO of the hip because of central nervous system injury, brain or spinal cord trauma, with functional limitations for daily activity and surgical indication for partial or total excision of heterotopic ossifications, were included. The surgical indications were major deformities with flessum, joint limitation, pain, and limited rehabilitative care due to NHO [3]. Patients with contra-indication to CT or MRI, such as pacemaker or metallic foreign body in the eye, major deformations preventing correct positioning on the examination table, or contra-indication to iodinated contrast agents, such as renal disease or history of reactions to contrast agents, were excluded as well as those without signed consent.

### CT technique

Patient installation was often difficult because of deformities and sometimes required use of cushions. Tourniquets were placed around thighs and calves in order to obtain better opacification of the main vessels rather than superficial vessels.

CT was performed with a scanner (SOMATOM Definition AS, Siemens Healthineers) after a biphasic iodinated solution injection (iomeprol, Iomeron**®** 400 mg/ml, Bracco Imaging) including a first injection of 120 ml at the rate of 1.5 ml/s immediately followed by a second injection of 80 ml at the rate of 3 ml/s. CT acquisition was performed with a collimation of 128 × 0.625 mm and was triggered 135 s after the start of injection. Voltage was 120 kV and amperage 300 mAs/slice. Rotation time was 0.5 s/rotation.

Multiplanar reconstructions were performed as well as bone and vessels volume rendering 3D images.

### MRI technique

Patient installation was crucial for the success of the exam. Cushions were used if necessary to facilitate the patient positioning. MRI was performed with a 3 T MR system (Discovery™ MR750 3.0 T, General Electrics). Protocol included:

1. 3D coronal spin-echo T1-weighted sequence with minimum echo time (TE), repetition time (TR) = 458 ms, echo train length (ETL) = 30, field of view (FOV) = 42.0, pixel size = 0.8 × 0.8 mm^2^, slice thickness = 1.6 mm, matrix = 512 × 512, bandwidth (BW) = 83.33 kHz, HyperSense (HS) = 1.5, number of excitation (NEX) = 1),
2. 3D axial spin-echo STIR sequence (TE = 110 ms, TR = 2800 ms, ETL = 110, TI = 210 ms, FOV = 42.0, pixel size = 1.4 × 1.4 mm^2^, slice thickness = 1.4 mm, matrix = 300 × 300, BW = 41.67 kHz, HS = 1.3, NEX = 1),
3. 3D axial ZTE sequence (FOV = 36.0, pixel size = 1.1 × 1.1 mm^2^, slice thickness = 1.6 mm, matrix/frequency = 320, spokes = 512, Flip Angle = 1°, NEX = 3, BW = 62,5 kHz).

Multiplanar and 3D volumic reconstructions of bone were performed with an Advantage Windows workstation v4.6 (General Electrics).

### Data reports

CT and MRI were analyzed by one radiologist with 4 years experience with pre-surgical bone assessment of NHO. To avoid any bias, there was 1 month interval between interpretation of each exam. A standardized report was completed for both CT and MRI for all patients. For each NHO, locations (anterior, posterior, inferior or external), circumferential extension, implantations (anterior border of the ilium, gluteal surface of the ilium, ilio-pubic branch, ischio-pubic branch, greater trochanter, lesser trochanter or femoral diaphysis), fragmentation (mono- or multi-fragmented), eventual pseudarthrosis, density (homogeneous or heterogeneous), relation to the joint capsule (no involvement, contact or disruption) and joint space (normal, narrowed or ankylosed) were analyzed.

**Fig. 1:**
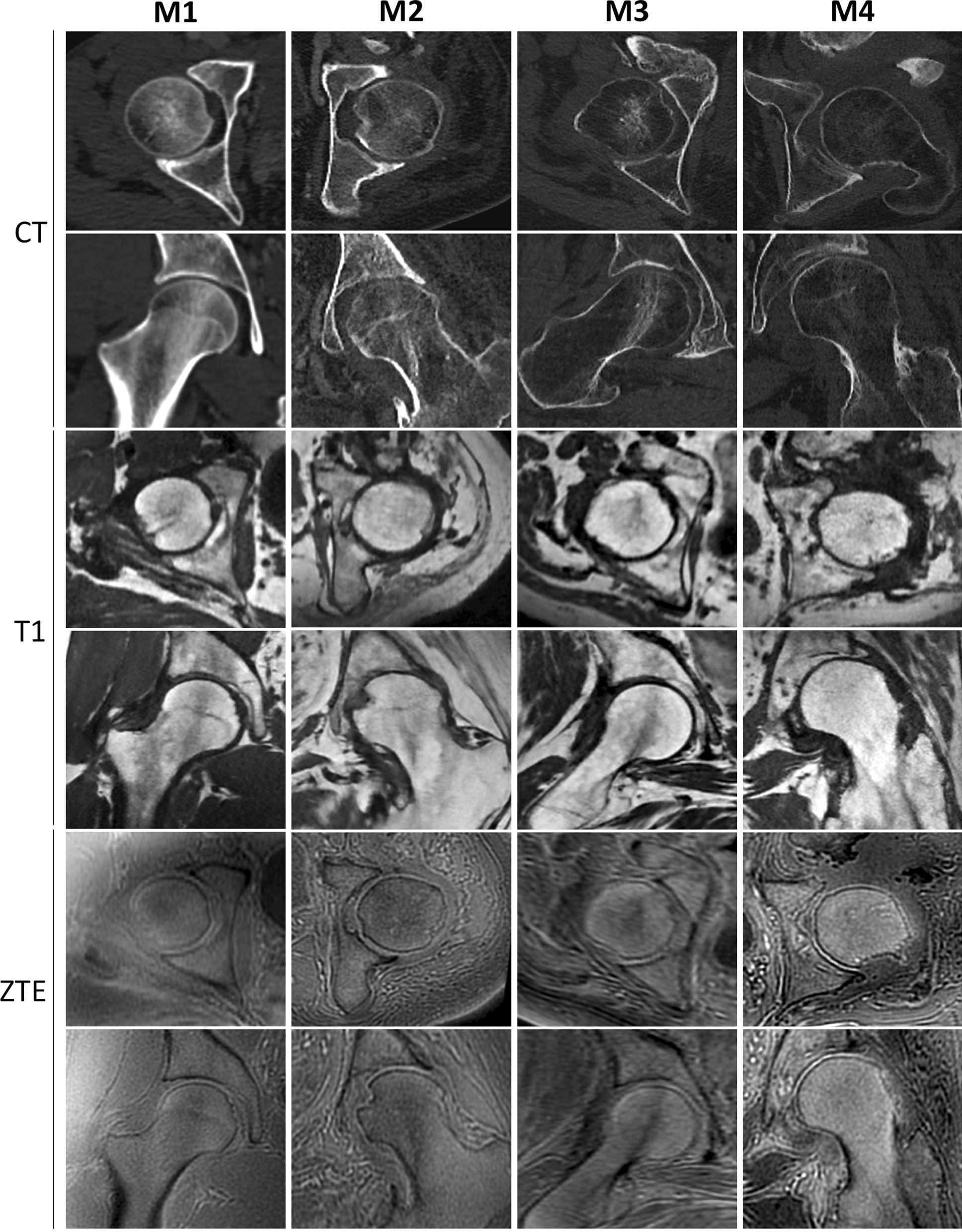
Qualitative evaluation of bone mineralization on CT and on MRI in T1-weighted and ZTE sequences, axial and coronal views of the femoral head. Each column shows a category of bone mineralization: M1, M2, M3 and M4

Qualitative evaluation of bone mineralization was also depicted and classified into four categories: normal (M1), mild demineralization (M2), significant demineralization (M3) and severe demineralization, with a replacement of osseous tissue by fatty tissue (M4). Analysis of bone mineralization on CT depended on the bone mineral density of the femoral head comparatively to the ilium [4]: similar density of the bone marrow of the femoral head to that of the ilium (M1); subcortical areas of punctuated hypodensities not seen in the ilium (M2); demineralization of the subcortical area of the femoral head with persistent central trabecular network (M3); disappearance of the trabecular bone of the femoral head which is totally replaced by fatty tissue while ilium is normal (M4). Analysis of bone mineralization on MRI depended on the signal intensity of femoral head on T1-weighted sequence and its trabecular bone density on T1-weighted and ZTE sequences. A classification corresponding to those described on CT [4] was established (Fig. 1): iso-intensity to diaphysis bone marrow on T1-weighted sequence and normal trabecular bone density (M1); moderate hyperintensity with subcortical areas of trabecular bone rarefaction (M2); subcortical hyperintensity with disappearance of the subcortical trabecular bone (M3); hyperintensity similar to subcutaneous fat with disappearance of the central and subcortical trabecular bone (M4).

Femoral neck fracture or osteonecrosis of the femoral head were reported if necessary.

Finally, surgical reports and hospitalization data were studied for per-operative or post-surgical complications. Post-surgical CT imaging was not systematically planned unless in case of complication.

### Statistical analysis

Time between pre-surgical imaging and surgery and ages are reported as “means ± standard deviation” and their ranges. Compared data between MRI and CT are expressed as observed concordance rates for each item, in percentage with corresponding exact ratio. A ratio < 0.40 was considered a moderate concordance, from 0.41 to 0.60 a moderate concordance, from 0.61 to 0.80 a good concordance and from 0.81 to 1.00 an excellent concordance.

## Results

### Population

From July 2019 to March 2020, 7 patients were included for surgical resection of NHO of the hip. Their ages ranged from 32 to 70 (mean = 50.2 ± 17.2 years). NHO were bilateral in 2 patients (29%), representing a total of 9 hips analyzed. Patients’ characteristics are described in Table 1.

**Table 1:** Patients’ characteristics

	Number (%)
Sex	
Male	5 (71%)
Female	2 (29%)
Age (years)^a^	50.2 ± 17.2
Etiology	
Brain trauma	2 (29%)
Spinal cord injury	4 (57%)
Stroke	1 (14%)
Hip	
Right	6 (67%)
Left	3 (33%)

^a^Ages are reported as mean ± standard deviation

Mean time between CT and surgery was 4.2 ± 3.3 months (0–230 days). Mean time between MRI and surgery was 5.9 ± 11.1 days (1–33 days), most often performed the day before the surgery.

### Analysis of the NHO

**Fig. 2:**
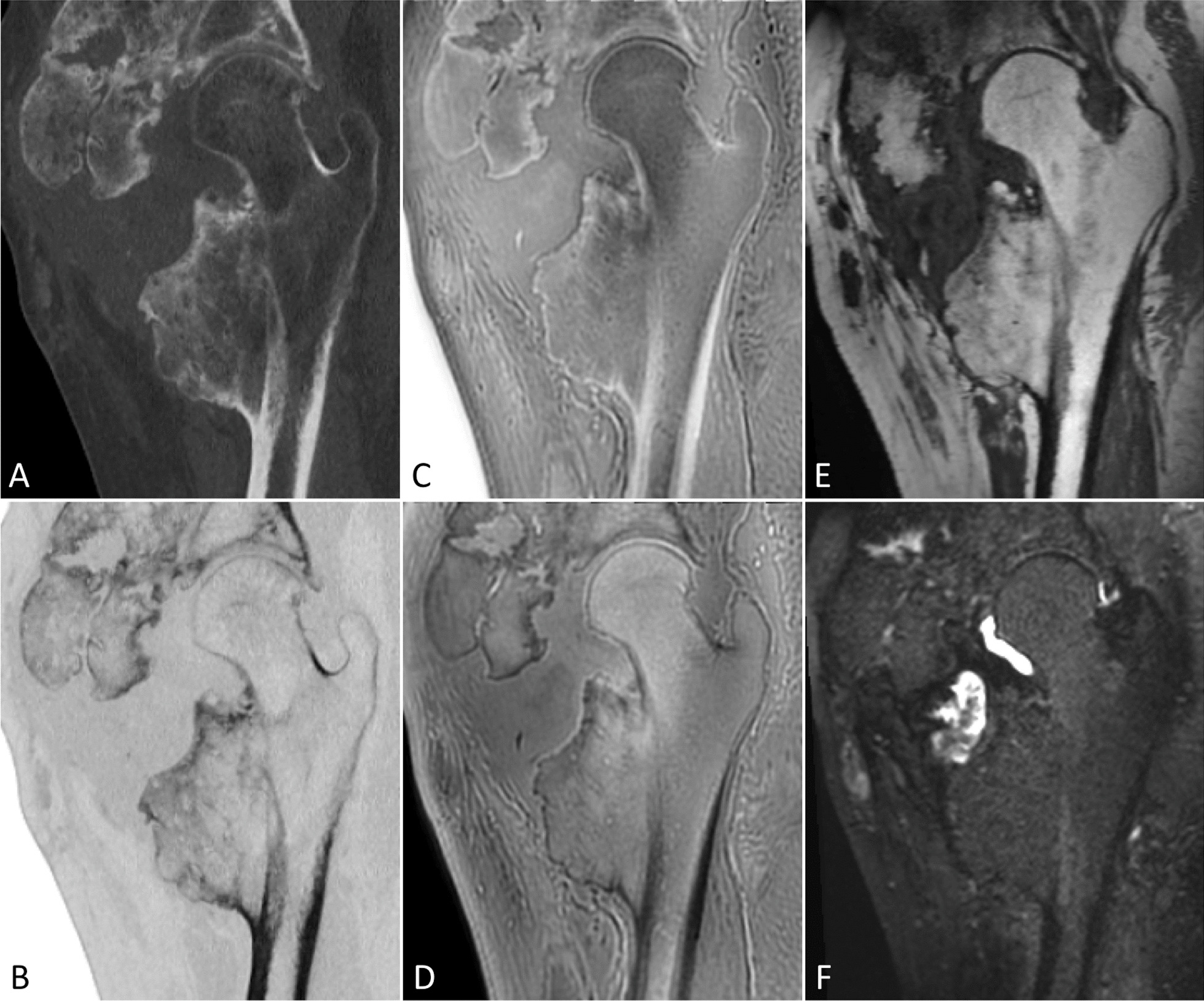
Heterogeneous multifragmented NHO of the right hip with pseudarthrosis. Comparative sagittal images in gray scale (**A**) and inverted gray scale (**B**) on CT, ZTE sequence with inverted gray scale (**C**), ZTE sequence (**D**), T1-weighted (**E**) and STIR sequences (**F**) on MRI. Note the articular effusion in the pseudarthrosis visible on the STIR sequence

NHO location was anterior or antero-inferior in 6 hips and posterior in 1 hip. A circumferential extension (anterior, posterior, and inferior) was found in 2 hips. The most frequent implantations were the anterior border of the ilium (8 hips), the greater trochanter (4 hips) and the femoral diaphysis (6 hips). NHO were homogeneous in 5 hips and heterogeneous in 4 hips. They were multifragmented in 8 hips and monofragmented in 1 hip. Pseudarthrosis was reported in 6 hips (Fig. 2).

**Fig. 3:**
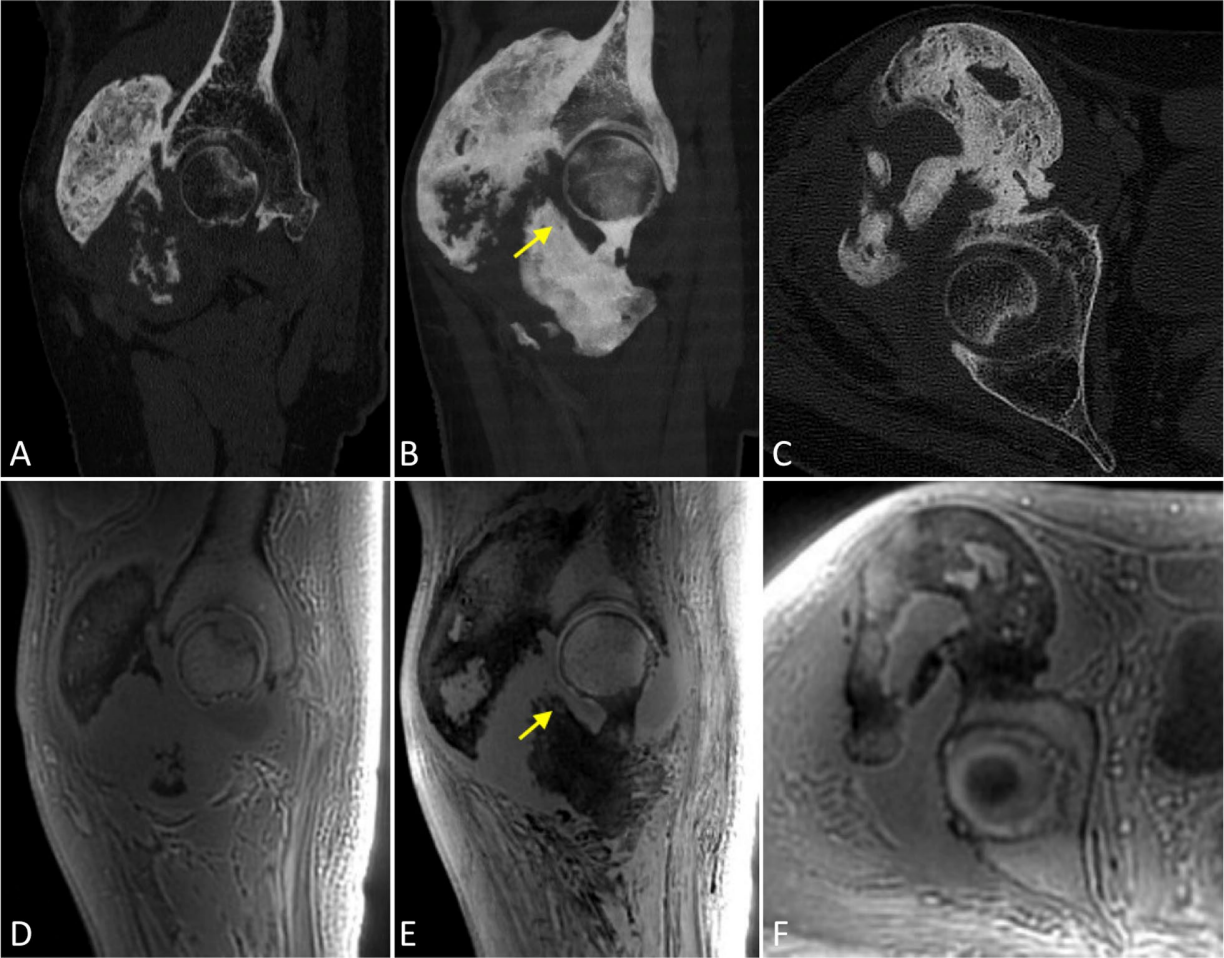
Voluminous anterior NHO oh the right hip with contact between the heterotopic ossifications and the joint capsule (arrow). Sagittal (**A**, **B**) and axial (**C**) CT images and their corresponding images on MRI in ZTE sequence (**D**–**F**)

Joint capsule was respected in all patients but a contact between heterotopic ossifications and the capsule was found in 7 hips (Fig. 3). Joint space was normal in 8 hips and narrowed in 1 hip. No patient had coxo-femoral joint ankylosis.

Bone mineralization was studied as well on CT as on MRI. Bone mineralization was normal (M1) in 1 hip. A mild demineralization (M2) was reported the most frequently, in 4 hips. Significant mineralization (M3) was found in 3 hips, and severe demineralization (M4) in 1 hip.

**Fig. 4:**
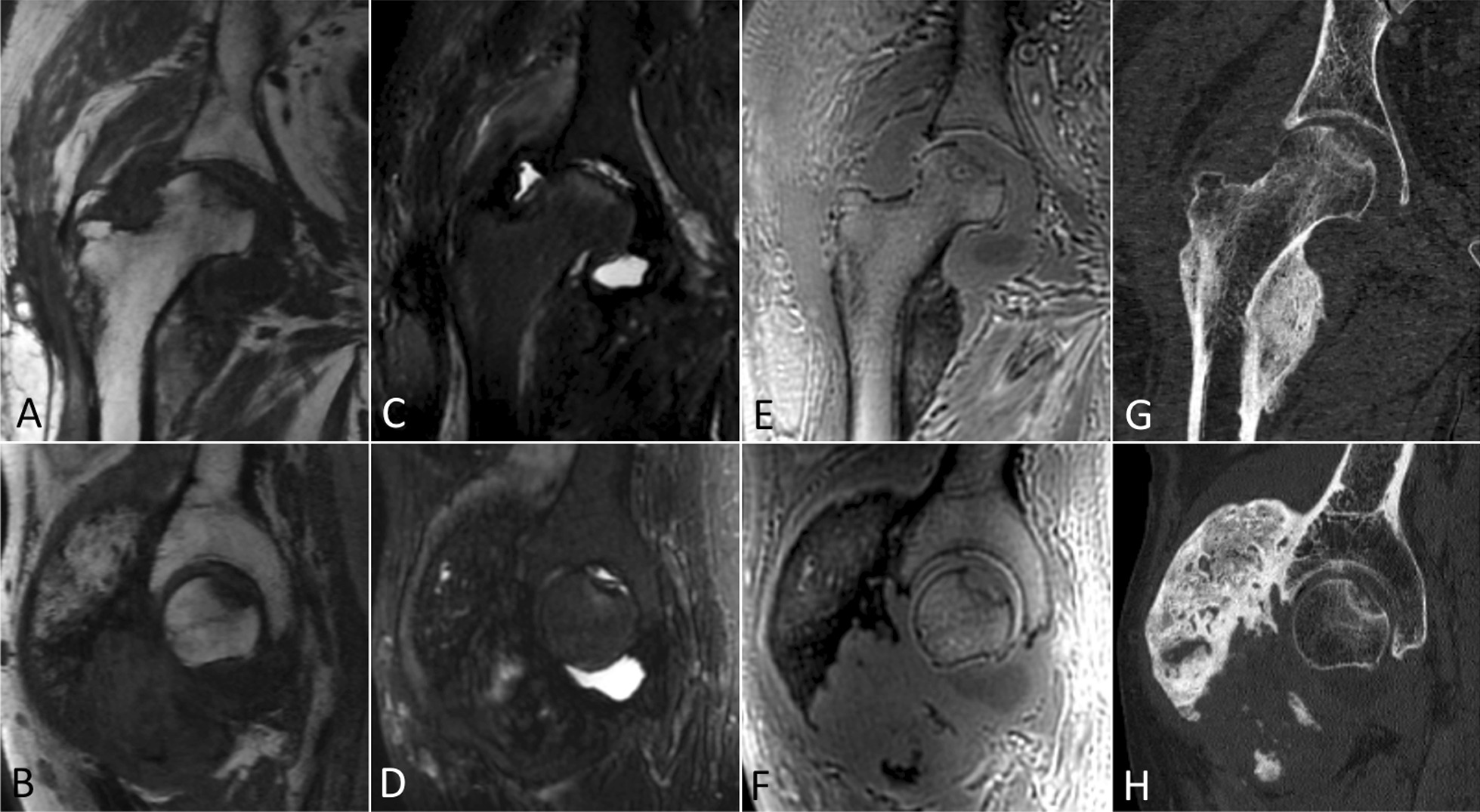
Osteonecrosis of the femoral head in a patient with an anterior NHO of the right hip. Coronal and sagittal images in T1-weighted (**A**, **B**), STIR (**C**, **D**), and ZTE (**E**, **F**) sequences and in CT (**G**, **H**)

Osteonecrosis of the femoral head was reported in one patient with NHO, diagnosed both on MRI and CT (Fig. 4). No patient showed femoral neck fracture.

Data are resumed in Table 2.

**Table 2:** Characteristics of the NHO

	Number (%)
Location	
Anterior	8 (89%)
Posterior	3 (33%)
Inferior	3 (33%)
External	1 (11%)
Circumferential extension	2 (22%)
Implantation	
Anterior border of the ilium	8 (89%)
Gluteal surface of the ilium	1 (11%)
Ilio-pubic branch	4 (44%)
Ischio-pubic branch	2 (22%)
Greater trochanter	4 (44%)
Lesser trochanter	3 (33%)
Femoral diaphysis	6 (67%)
Fragmentation	
Monofragmentary	1 (11%)
Multifragmentary	8 (89%)
Pseudarthrosis	6 (67%)
Density	
Homogeneous	5 (56%)
Heterogeneous	4 (44%)
Borders	
Sharp	5 (56%)
Ill-defined	4 (44%)
Relation to joint capsule	
No involvment	2 (22%)
Contact	7 (78%)
Disruption	0 (0%)
Joint space	
Normal	8 (89%)
Narrowed	1 (11%)
Ankylosis	0 (0%)
Bone mineralization	
Normal (M1)	1 (11%)
Mild demineralization (M2)	4 (45%)
Significant demineralization (M3)	3 (33%)
Severe demineralization (M4)	1 (11%)
Femoral neck fracture	0 (0%)
Osteonecrosis of the femoral head	1 (11%)

### Concordance between CT and MRI (Table 3)

**Table 3:** Comparison of MRI and CT data

No of patient and modality		Location	Circum-ferential extension	Implantation	Fragmentation	Pseud-arthrosis	Density	Borders	Relation to the joint capsule	Joint space	Bone minera-lization	Fracture /osteo-necrosis
1	CT	P	No	ABI GT	Multifragmentary	Yes	Homogeneous	***Ill-defined***	***Contact***	Normal	***M3***	No/No
	MRI	P	No	ABI GT	Multifragmentary	Yes	Homogeneous	***Sharp***	***No involvment***	Normal	***M2***	No/No
2	CT	A P I	Yes	ABI IlPB LT	Multifragmentary	Yes	Homogeneous	***Ill-defined***	Contact	Normal	***M2***	No/No
	MRI	A P I	Yes	ABI IlPB LT	Multifragmentary	Yes	Homogeneous	***Sharp***	Contact	Normal	***M3***	No/No
3	CT	A	No	ABI GT FD	Multifragmentary	Yes	Heterogeneous	Sharp	Contact	Normal	M3	No/Yes
	MRI	A	No	ABI ***GSI*** GT ***LT*** FD	Multifragmentary	Yes	Heterogeneous	Sharp	Contact	Normal	M3	No/Yes
4	CT	A ***I***	No	ABI IsPB ***GT***	Monofragmentary	No	Homogeneous	***Ill-defined***	Contact	Narrowed	M3	No/No
	MRI	A	No	ABI IsPB ***LT FD***	Monofragmentary	No	Homogeneous	***Sharp***	Contact	Narrowed	M3	No/No
5	CT	A	No	ABI IlPB GT ***LT*** FD	Multifragmentary	Yes	Heterogeneous	Sharp	No involvment	Normal	***M3***	No/No
	MRI	A	No	ABI IlPB GT FD	Multifragmentary	Yes	Heterogeneous	Sharp	No involvment	Normal	***M2***	No/No
6	CT	A ***I***	No	IsPB FD	***Multifragmentary***	***Yes***	***Homogeneous***	***Sharp***	***Contact***	Normal	M2	No/No
	MRI	A	No	IsPB FD	***Monofragmentary***	***No***	***Heterogeneous***	***Ill-defined***	***No involvment***	Normal	M2	No/No
7	CT	A	No	ABI ***LT*** FD	Multifragmentary	Yes	Homogeneous	Sharp	Contact	Normal	M2	No/No
	MRI	A	No	ABI FD	Multifragmentary	Yes	Homogeneous	Sharp	Contact	Normal	M2	No/No
8	CT	A P E I	Yes	ABI GSI IlPB LT FD	Multifragmentary	No	Heterogeneous	Sharp	Contact	Normal	M4	No/No
	MRI	A P E I	Yes	ABI GSI IlPB LT FD	Multifragmentary	No	Heterogeneous	Sharp	Contact	Normal	M4	No/No
9	CT	A	No	ABI IlPB LT ***FD***	Multifragmentary	No	***Heterogeneous***	Ill-defined	No involvment	Normal	M1	No/No
	MRI	A	No	ABI IlPB LT	Multifragmentary	No	***Homogeneous***	Ill-defined	No involvment	Normal	M1	No/No

Discordances between CT and MRI reports are exposed in bold and italic

*A* anterior, *P* posterior, *E* external, *I* inferior, *ABI* anterior border of the ilium, *GSI* gluteal surface of the ilium, *IlPB* ilio-pubic branch, *IsPB* ischio-pubic branch, *GT* greater trochanter, *LT* lesser trochanter, *FD* femoral diaphysis

Observed concordance rates between MRI and CT were 94.4% (34/36) for the location, 100% (9/9) for the circumferential extension, 87.3% (55/63) for the implantation, 88.9% (8/9) for the fragmentation, 77.8% (7/9) for the density.

Observed concordance rate was 77.8% (7/9) for the relation to the joint, 100% (9/9) for the joint space and 66.7% (6/9) for the qualitative evaluation of bone mineralization. It was 100% (9/9) for both femoral neck fracture and osteonecrosis of the femoral head.

Discordances between MRI and CT (also exposed in Table 3) were most common for bone mineralization and density evaluation, respectively 3/9 hips and 2/9 hips.

**Fig. 5:**
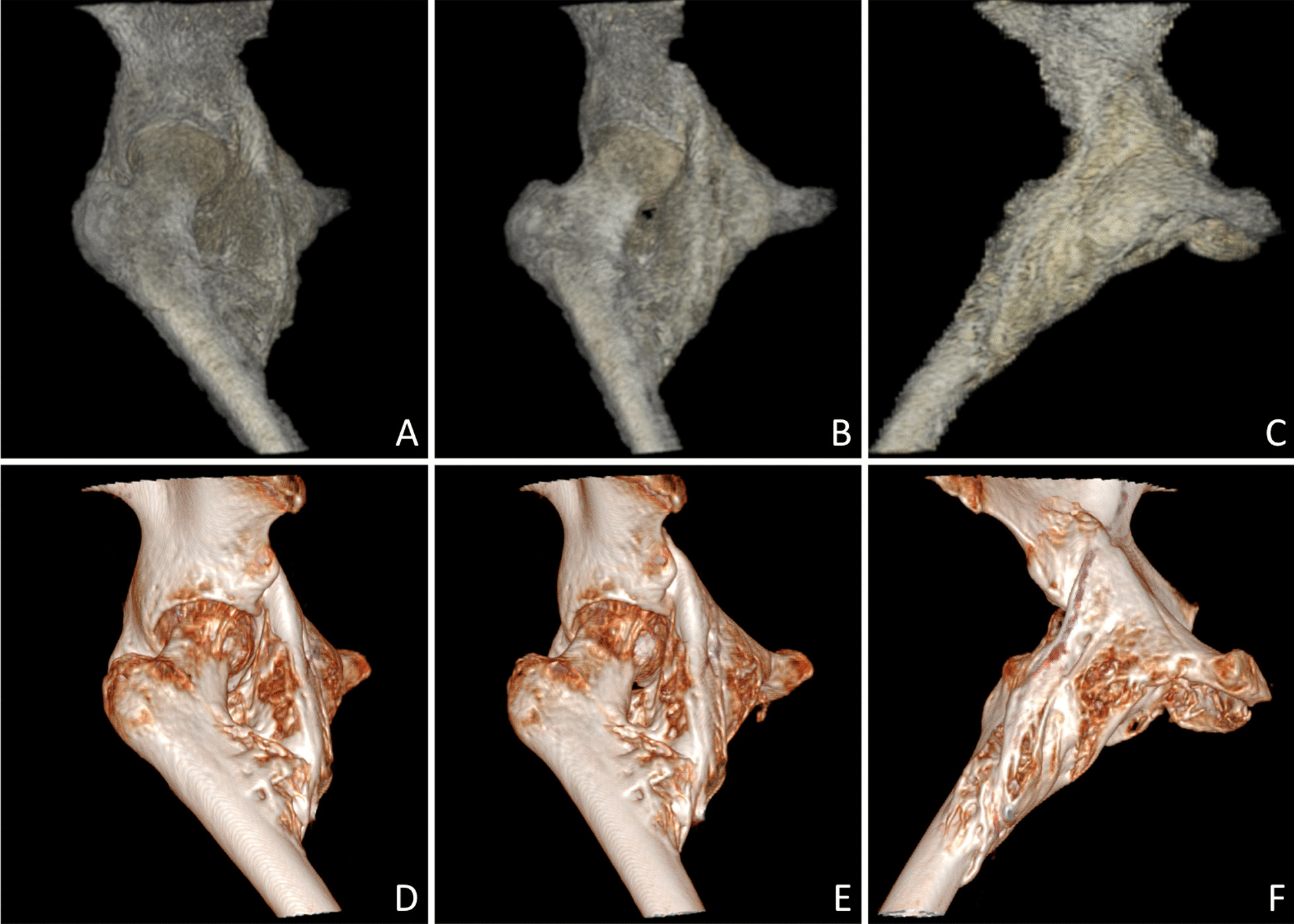
Antero-internal monofragmented NHO of the right hip. 3D volumic reconstructions on ZTE sequence (**A**–**C**) and their corresponding images on CT (**D**–**F**)

**Fig. 6:**
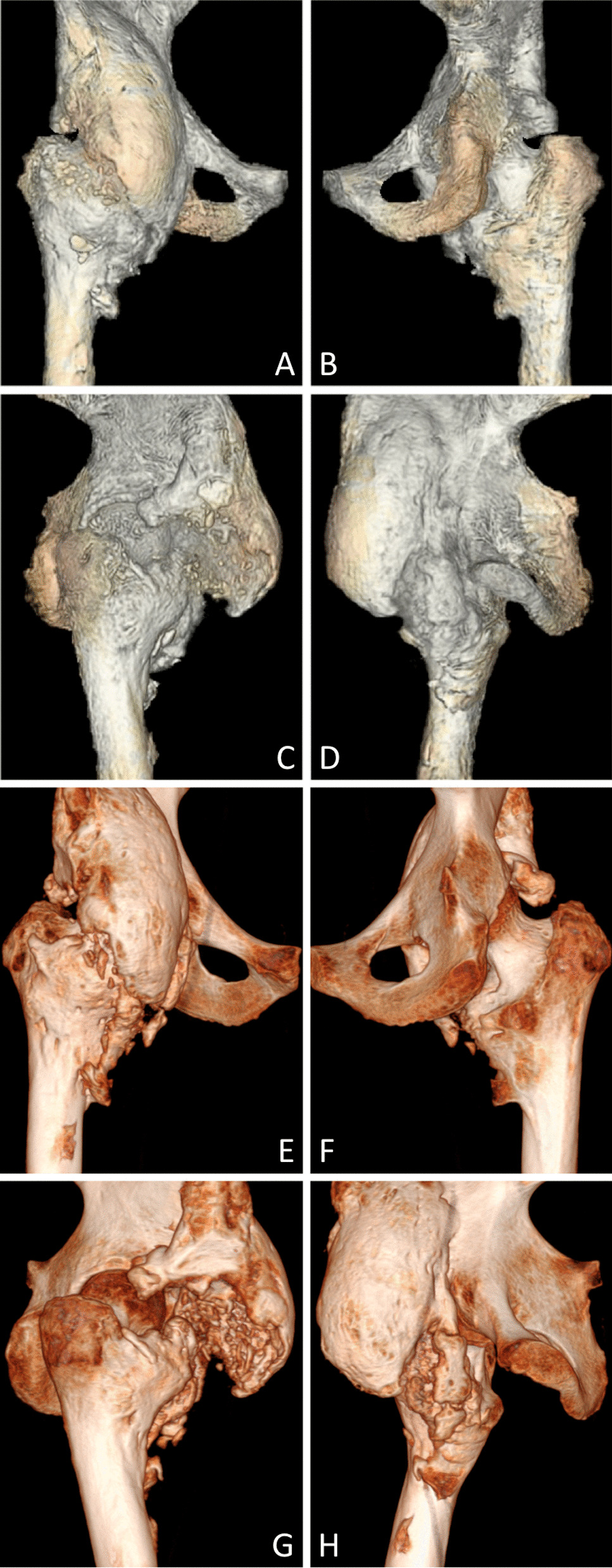
Anterior multifragmented NHO of the right hip. 3D volumic reconstructions with anterior, posterior, right and left views on ZTE sequence (**A**–**D**) and their corresponding images on CT (**E**–**H**)

3D volumic views appeared to be similar in both MRI and CT (Figs. 5, 6).

### Complications

For all the seven patients who were operated, no intra-operative complication such as vascular injury or fracture occurred. In one patient, a urinary infection occurred few days after surgery.

## Discussion

We reported an excellent agreement between MRI and CT for the heterotopic ossifications location and their features on pre-surgical assessment of NHO of the hip. In this prospective preliminary study, MRI and CT findings are compared for the first time in this indication.

MRI is a non-irradiating alternative to CT, particularly useful for hypersensitivity reactions to iodinated contrast or chronic renal failure. Thanks to morphological T1-weighted and STIR sequences, and more specifically to ZTE sequence, MRI provides excellent spatial resolution and high contrast. Bone analysis on MRI had been of low quality for a long time because relaxation time of bone tissue is 300–500 μs whereas relaxation times of gradient echo or spin echo sequences is 200–3000 ms. With ZTE sequence, images are acquired with echo time of as low as a few microseconds after radiofrequency excitation pulse, with pure frequency encoding in a 3D radial center-out k-space encoding scheme [8]*.* Then, bone structures, more specifically cortical bone, which are low protons dense, appear hypo-intense. On the contrary, soft tissues containing more protons appear iso- to hyperintense. Moreover, ZTE sequence is highly weighted in protons density and then soft tissues are not contrasted. That is why this sequence provides excellent contrast between cortical bone and soft tissues, as well as CT. It allows an accurate analysis of the heterotopic ossifications, their location, implantation and relation to the joint capsule.

However, 3D volumic reconstructions of ZTE sequences require a post processing software, including segmentation and manual contouring for each lesion, which is time consuming. Softwares improvements in segmentation and reconstruction have to be developed to obtain repeatable reconstructions for clinical routine use.

Bone mineralization is important on pre-surgical assessment of the hip, because high demineralization is associated with high risk of peri-operative femoral neck or epiphysis fracture [13]. Dual-energy X-ray absorptiometry (DXA) is not feasible because of the heterotopic ossifications. Bone mineralization is usually evaluated on CT by the bone density and the trabecular bone network. But this type of measurement is more suitable for vertebrae than for hips. A qualitative evaluation of bone mineralization and bone fracture has been proposed on CT in pre-surgical status and offer a simple and accurate tool to predict hip fracture in peri-operative period [4].

Qualitative bone mineralization analysis on MRI has been only few described. Its feasibility in vivo by quantifying bone water and mineral phosphorus with ZTE has been proved [14] but without clinical application because of too long MRI protocols. Quantification of fat fraction using T1 DIXON sequence or extrapolation of cellularity in bone marrow using Diffusion-Weighted Imaging (DWI) and apparent diffusion coefficient (ADC) are also available [15]. In this study, we propose a semi-quantitative classification, fast and repeatable, applicable in clinical routine as it does not require post-processing.

This MRI classification is based on the CT one, with the same semiological analysis. First, the amount of fat in the medullary bone in the femoral head on T1-weighted sequence, ranging progressively from iso-intensity to other medullary bone if normal (M1) to hyperintensity similar to subcutaneous fat (M4). Second, the trabecular bone density on T1-weighted and ZTE sequences, ranging from normal trabecular bone density (M1) to subcortical areas of trabecular bone rarefaction (M2) then disappearance of the subcortical trabecular bone with some central trabecular network preserved (M3) and ultimately central and subcortical trabecular bone (M4). This classification appears satisfying with an observed concordance rate of 77.8% between MRI and CT, but requiring to be carried out on more patients. It might be defeated in case of signal abnormality, particularly in case of acute femoral neck fracture or femoral head osteonecrosis.

Some discordances between MRI and CT were reported, especially for bone mineralization and borders evaluation. Most of them can be explained by subjective analysis of the heterotopic ossifications features whose differences are sometimes very small. Furthermore, pre-surgical MRI assessment of NHO is a new imaging technique which requires time adaptation for more reproducibility.

The current study has some limitations. First, this preliminary study was performed on only seven patients and requires to be carried out on more patients to confirm these results. Secondly, pre-surgical assessment of NHO requires vascular cartography for vessels which are near the ossifications. In this study, CT which is the gold standard was performed after a biphasic iodinated solution injection whereas MRI was realized without contrast injection. Indeed, the aim of this study was to compare MRI and CT to assess the heterotopic ossifications location and their features. Comparison of these imaging modalities for NHO relations to vessels will be the next step for evaluating the MRI as pre-surgical assessment of NHO of the hip.

The ZTE sequence is a new MRI sequence that requires adaptation time and training for the radiologist to be able to perform a correct analysis. If as we assume vascular analysis in MRI would be as effective as in CT, MRI should be a valuable alternative to CT in daily practice, particularly for patients with renal failure or allergy to iodinated contrast agents.

In conclusion, MRI provides excellent data on heterotopic ossifications, as well as CT, for pre-surgical assessment of NHO of the hip: location, circumferential extension, implantation, fragmentation, relation to the joint capsule, joint space and bone mineralization. Then, MRI could be a valuable alternative to CT if necessary. Comparison between MRI and CT should be continued, particularly for relations to vessels and nerves assessment, the weak point of CT being the nerves, muscular denervation and inflammation.

## Data Availability

The datasets used and/or analysed during the current study are available from the corresponding author on reasonable request.

## Abbreviations

NHO: Neurogenic heterotopic ossification
NMO: Neurogenic myositis ossificans
STIR: Short-tau inversion recovery
ZTE: Zero Echo Time
